# "Black Esophagus" or Gurvits Syndrome: A Rare Cause of Upper Gastrointestinal Bleeding in Diabetic Ketoacidosis

**DOI:** 10.7759/cureus.34989

**Published:** 2023-02-14

**Authors:** Fouad Jaber, Saqr Alsakarneh, Sruthi Sripada, Rishabh Gaur, Rawan Rajab, Islam Mohamed, Kimberly Sanders, Hassan Ghoz

**Affiliations:** 1 Internal Medicine, University of Missouri Kansas City, Kansas, USA; 2 Gastroenterology and Hepatology, University of Missouri Kansas City, Kansas, USA

**Keywords:** hematemesis, dka, gurvits syndrome, acute esophageal necrosis (aen), black esophagus

## Abstract

Black esophagus, also called Gurvits syndrome or acute esophageal necrosis (AEN), is a rare, life-threatening condition characterized by necrosis of the esophageal mucosa. We present a 36-year-old man who presented with hematemesis and was admitted for diabetic ketoacidosis (DKA) management. He then had a further episode of hematemesis with hemodynamic instability.

The esophagogastroduodenoscopy (EGD) revealed ulcerative, necrotizing, circumferential esophagitis in the middle and distal third of the esophagus. The patient was treated with intravenous fluid resuscitation, proton pump inhibitors, empiric antibiotics, and antifungals. Hematemesis in DKA should raise suspicion for black esophagus. Prompt detection of AEN allows for early management and thus reduces mortality and associated complications such as perforations and strictures.

## Introduction

Black esophagus, also known as Gurvits syndrome or acute esophageal necrosis (AEN), is a rare condition with an incidence of 0.1-0.28% [1] and is characterized by circumferential black discoloration of the esophageal mucosa on esophagogastroduodenoscopy (EGD) [2]. Typically, necrosis is seen in the distal esophagus but can also affect the proximal and middle third [2]. Comorbidities associated with a higher risk of developing black esophagus include diabetes mellitus, hypertension, kidney disease, alcoholism, malnutrition, and cardiovascular diseases [2]. Recently, black esophagus has been associated with diabetic ketoacidosis (DKA) [3-7]. We present a case of black esophagus associated with DKA presenting with hematemesis.

## Case presentation

A 36-year-old man with uncontrolled type 1 diabetes mellitus, end-stage renal disease on hemodialysis, and hypertension presented with abdominal pain, nausea, and vomiting. He had hemodialysis the day before admission. The patient reported progressive, generalized, constant, non-radiating epigastric pain and vomiting 200-300 mL of bright red blood. He denied melena and hematochezia. No use of tobacco, alcohol, recreational drugs, or non-steroidal anti-inflammatory drugs (NSAIDs). No history of liver disease and no prior esophagogastroduodenoscopy (EGD). On arrival, the patient was hemodynamically stable. Physical examination revealed normoactive bowel sounds and a non-distended abdomen with generalized tenderness but no peritoneal signs. Arterial blood gas analysis showed a pH of 7.3, bicarbonate of 9 mmol/L, and anion gap of 41 mmol/L. Complete blood count showed a hematocrit of 29%, a white blood cell count of 10.2 x 10^9/l, a hemoglobin of 7.5 g/dl, a platelet count of 148 x 10^9/l, a prothrombin time (PT) of 12.4 s, and an international normalized ratio (INR) of 1.1. Blood chemistry was notable for glucose of 1491 mg/dL, blood urea nitrogen (BUN) of 101 mg/dL, creatinine of 6.32, potassium of 7.2 mmol/L, lipase of 41 U/L, lactic acid of 1.4 mmol/L, and sodium of 121 mmol/l. The patient was admitted to the critical care unit for DKA treatment with insulin infusion and intravenous (IV) fluid resuscitation.

**Figure 1:**
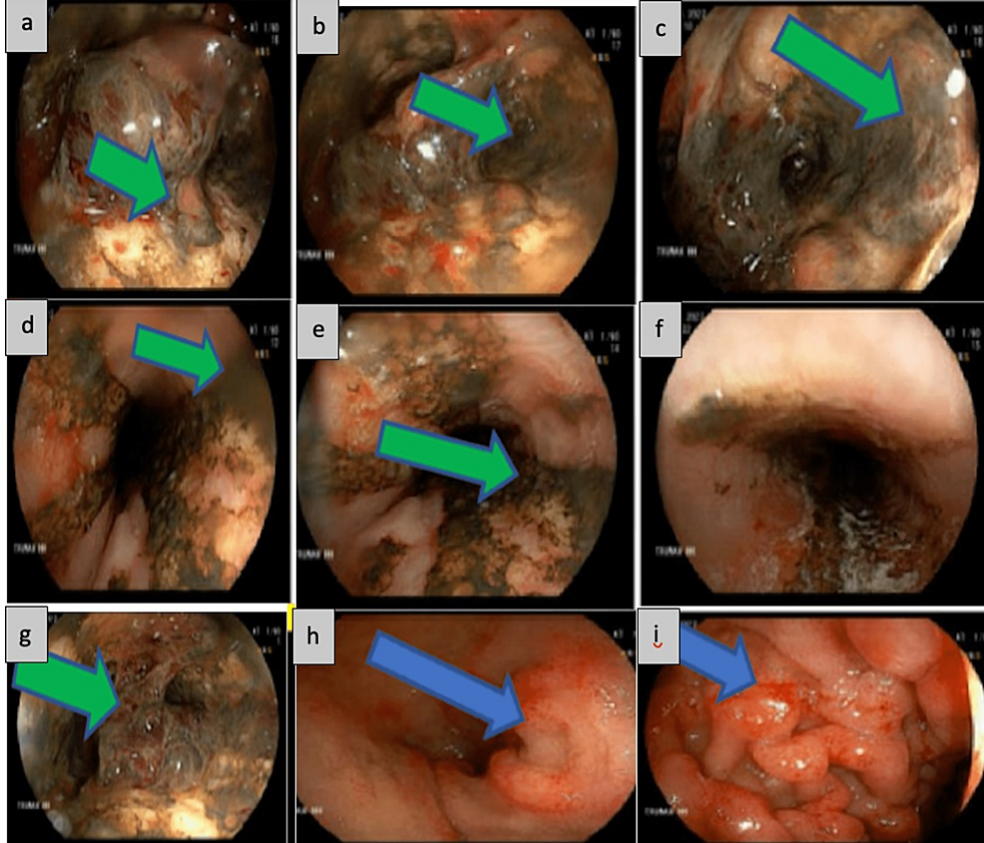
Esophagogastroduodenoscopy (EGD) EGD images showing severe, ulcerative, necrotic, circumferential esophagitis in the middle and lower third esophagus (green arrows). Just above the gastroesophageal junction, a medium-sized blood clot was seen (blue arrows) without evidence of active bleeding. The area adjacent to the clot beyond the depth of insertion was suspicious for deep mucosal tears and possible false lumen.

The following day, the patient had an episode of hematemesis with a heart rate of 110 beats per minute, a blood pressure of 95/54 mmHg, and a hemoglobin level that had fallen to 5.6 g/dL. The patient was transfused and started on pantoprazole infusion with improvement in vital signs and hemoglobin. Esophagogastroduodenoscopy (EGD) (Figure 1) showed severe, ulcerative, necrotizing, circumferential esophagitis in the middle and distal third of the esophagus, beginning 20 cm from the incisors, consistent with black esophagus. A medium-sized blood clot was found just above the gastro-esophageal junction (GEJ) with no evidence of active bleeding. The area adjacent to the clot beyond the depth of insertion was suspicious for a deep mucosal tear and possible false lumen. Thus, the clot was not manipulated, minimal air insufflation was used, and no biopsy was taken.

A chest computed tomography (CT) with contrast showed no evidence of esophageal perforation. After EGD, the patient had several small, self-limiting episodes of hematemesis and melena but remained hemodynamically stable. Pantoprazole infusion was continued, and he received empiric antimicrobial therapy with ampicillin-sulbactam and fluconazole for five days. The patient had no further episodes of hematemesis but had light melanotic stools. His hemoglobin stabilized at about 8 g/dl, and he was discharged from the hospital on Day 7. He was lost to follow-up at the EGD outpatient department. 

## Discussion

Black esophagus is a life-threatening condition with multi-factorial etiologies [2]. However, the primary hypothesized pathophysiological mechanism is hypoperfusion or compromised mucosal barrier susceptible to acid reflux [1,2]. Such damage to the mucosa is commonly found in the distal esophagus due to known watersheds causing decreased vascularity of the area [1,2]. Low perfusion states are critical to the presentation and development of acute esophageal necrosis (AEN), as patients often present with other comorbidities such as sepsis, congestive heart failure, renal failure, pancreatitis, hypothermia, and shock [2,3,7]. Black esophagus has been reported in a few cases in association with diabetic ketoacidosis (DKA) [3-10]. In particular, a literature review showed a strong association between DKA and AEN in acute gastrointestinal bleeding [10]. The exact mechanism is not fully understood but few have been suggested. Hyperglycemia in DKA leads to poor vascular flow and sustainable damage to the esophageal mucosal barrier [7, 11]. Similarly, osmotic diuresis in DKA causes extreme volume depletion and hypoperfusion of the distal esophagus, resulting in ischemia and necrosis of the area [7,10,11]. Other associated comorbidities, end-stage renal disease in our case, can increase the risk and severity of AEN [2].

Common manifestations of AEN include epigastric abdominal pain, hematemesis, nausea, and melena [2,7,10]. Esophagogastroduodenoscopy (EGD) is usually required to diagnose AEN because it allows direct visualization of black necrotic areas and esophagitis [2,10]. In addition, EGD is necessary to detect complications such as esophageal perforation (< 7%) and stricture (> 10%), which are potentially severe complications of AEN [8,10]. When possible, a biopsy can be taken to rule out bacterial, viral, or fungal infections, which are commonly associated with AEN [2,3,11]. While no histological examination is required for diagnosis, biopsy results distinguish between possible causes of black esophagus, thus guiding management [3,8,10]. Biopsy and histological examination of the affected region typically show inflammation and necrosis extending through the entire thickness of the esophageal wall [3,8,10]. A biopsy was not pursued in our case due to the risk of perforation with a suspected deep mucosal tear and possible false lumen.

The main goal in the treatment of AEN is resuscitation with intravenous fluids, blood transfusion, and proton pump inhibitors infusion to control gastric acidity and protect against further upper gastrointestinal bleeding [10-12]. Infections are common in conditions with low perfusion such as AEN [2,8,10]. Empiric antibiotics have been used in many reported cases of AEN empirically or if there is a concomitant infection or concerns about esophageal perforation [2,10]. Antibiotics can be tailored later based on culture and susceptibilities. In addition, other authors suggest the concomitant empirical use of antifungals [3,12-14]. The mortality rate of black esophagus is about 36%, with a higher risk in case of complications such as strictures and esophageal perforations [2,10]. 

## Conclusions

To conclude, acute esophageal necrosis (AEN) should be considered in patients with diabetic ketoacidosis (DKA) presenting with upper gastrointestinal bleeding. Esophagogastroduodenoscopy (EGD) is diagnostic, and it is recommended that a biopsy be obtained when possible to confirm the diagnosis unless the risk of perforation is high. Empiric antibiotics with or without antifungals should be considered in case of concomitant evidence of infection or perforation. Prompt detection is crucial to avoid undesired complications such as strictures and perforations.
